# Genome-wide gene by lead exposure interaction analysis identifies *UNC5D* as a candidate gene for neurodevelopment

**DOI:** 10.1186/s12940-017-0288-3

**Published:** 2017-07-28

**Authors:** Zhaoxi Wang, Birgit Claus Henn, Chaolong Wang, Yongyue Wei, Li Su, Ryan Sun, Han Chen, Peter J. Wagner, Quan Lu, Xihong Lin, Robert Wright, David Bellinger, Molly Kile, Maitreyi Mazumdar, Martha Maria Tellez-Rojo, Lourdes Schnaas, David C. Christiani

**Affiliations:** 1 Harvard TH Chan School of Public Health, Boston, MA USA; 2 Department of Environmental Health, Boston University, School of Public Health, Boston, USA; 3 Genome Institute of Singapore, Singapore, Singapore; 4 Department of Epidemiology, Department of Biostatistics, School of Public Health, Nanjing Medical University, Nanjing, China; 5 Mt. Sinai School of Medicine, New york, USA; 6 Children’s Hospital Boston, Boston, USA; 7 Oregon State University, Corvallis, USA; 8 Instituto Nacional de Salud Publica, Cuernavaca, Mexico; 9 Instituto Nacional de Perinatologia, Mexico City, Mexico

**Keywords:** Genome-wide association study, Gene-environment interactions, Child development, Lead poisoning, Single nucleotide polymorphism, UNC5D, SLC1A5, Environmental health

## Abstract

**Background:**

Neurodevelopment is a complex process involving both genetic and environmental factors. Prenatal exposure to lead (Pb) has been associated with lower performance on neurodevelopmental tests. Adverse neurodevelopmental outcomes are more frequent and/or more severe when toxic exposures interact with genetic susceptibility.

**Methods:**

To explore possible loci associated with increased susceptibility to prenatal Pb exposure, we performed a genome-wide gene-environment interaction study (GWIS) in young children from Mexico (*n* = 390) and Bangladesh (*n* = 497). Prenatal Pb exposure was estimated by cord blood Pb concentration. Neurodevelopment was assessed using the Bayley Scales of Infant Development.

**Results:**

We identified a locus on chromosome 8, containing *UNC5D*, and demonstrated evidence of its genome-wide significance with mental composite scores (rs9642758, *p*_meta_ = 4.35 × 10^−6^). Within this locus, the joint effects of two independent single nucleotide polymorphisms (SNPs, rs9642758 and rs10503970) had a *p*-value of 4.38 × 10^−9^ for mental composite scores. Correlating GWIS results with in vitro transcriptomic profiles identified one common gene, *SLC1A5,* which is involved in synaptic function, neuronal development, and excitotoxicity. Further analysis revealed interconnected interactions that formed a large network of 52 genes enriched with oxidative stress genes and neurodevelopmental genes.

**Conclusions:**

Our findings suggest that certain genetic polymorphisms within/near genes relevant to neurodevelopment might modify the toxic effects of Pb exposure via oxidative stress.

**Electronic supplementary material:**

The online version of this article (doi:10.1186/s12940-017-0288-3) contains supplementary material, which is available to authorized users.

## Background

Human development is a trajectory reflecting dynamic processes mediated by constant interplay between genetic background and environmental exposures. Toxic chemicals can alter this trajectory to produce maladaptive cognitive and behavioral phenotypes. Although only a small subset of children exposed to neurotoxicants present a clinically defined developmental disorder, [1], many more exposed individuals probably exhibit subclinical effects, such as reduced IQ. Variability in outcome with the same levels of observed exposures may be explained, in part, by genetic variability that can alter susceptibility to a neurotoxicant. While cognitive development has genetic contributions [2], multiple genome-wide association studies (GWAS) have failed to identify common variants that alone explain the variance in cognition [3–5]. Interactions between genetic and environmental factors may account for a large proportion of the variance and may provide useful information on toxicant pathogenesis [6]. In a cohort of Mexican mother-infant pairs, apolipoprotein E genotypes are associated with 24-month Mental Development Index (MDI) of the Bayley Scale, which might modify lead neurotoxicity [7].

The weight of evidence supports an association between early life exposure to lead (Pb) and impaired cognitive function in children [8–10]. The central nervous system (CNS) is highly vulnerable to chemical injury during development because critical processes—including neuronal growth, synaptic network formation, neuronal migration, and receptor development—peak during this time [11, 12]. Previous studies have shown that Pb crosses the placenta and accumulates in fetal tissues [13, 14]. While the health effects of early Pb exposure are well described, it remains unclear whether certain individuals are genetically predisposed to adverse outcomes following Pb exposure, as few studies have examined gene–environment (GxE) interactive effects on neurodevelopment. This knowledge is central to understanding the mechanisms of action and to creating effective interventions for prevention and treatment of toxic exposures.

The objective of our study was to examine GxE interactions on neurodevelopmental outcomes in two populations exposed to Pb. We examined prenatal exposure because many genes are expressed only during that specific developmental stage, and also because prenatal exposure has consistently been associated with adverse neurodevelopmental outcomes [15, 16]. Thus, genetic variants that produce GxE interactions may only do so when the exposure timing corresponds to a critical developmental window during which the relevant gene is highly expressed. We conducted a genome-wide GxE interaction study (GWIS) in two birth cohorts of Mexican and Bangladeshi children to identify genetic loci that modify the effect of prenatal Pb exposure on neurodevelopmental scores at approximately 2 years of age. We also performed integrative analyses using in vitro transcriptomic data of Pb-induced changes in human neural stem cells (hNSCs).

## Methods

### Study populations

#### Mexico cohort

Between 2007 and 2011, we enrolled participants in the Programming Research in Obesity, Growth, Environment and Social Stressors (PROGRESS) study, a prospective birth cohort to evaluate environmental and social stressors in relation to child growth and development. Recruitment and enrollment have been described previously [17]. Briefly, we recruited healthy pregnant women between 12 and 24 weeks gestation who were living in Mexico City. Women were enrolled from prenatal clinics and maternity hospitals affiliated with the Instituto Mexicano del Seguro Social, the Mexican social security system. The Instituto Mexicano del Seguro Social offers healthcare to private sector, middle-class workers and their families and provides coverage to 34.3% of Mexico City residents (according to 2010 National Census). Eligible participants were at least 18 years of age, had access to a telephone, lived within the Mexico City metropolitan area, and planned to remain there for 3 years. Exclusion criteria included history of heart or kidney disease, daily alcohol consumption, and use of steroids or antiepilepsy drugs. A total of 1054 women were informed about the study and provided written consent to participate. Participating women gave birth to 948 singleton infants, of which 541 were followed until 2 years of age and were available for neurodevelopmental assessment. The human subject committees of the National Institutes of Public Health in Mexico, Harvard T.H. Chan School of Public Health, Icahn School of Medicine at Mt. Sinai, and participating hospitals approved all study materials and procedures.

#### Bangladesh cohort

Between 2008 and 2011, we recruited pregnant women to participate in a prospective reproductive health cohort to examine the effects of chronic low-level arsenic exposure on reproductive and neurologic health outcomes. As described previously [18], pregnant women were recruited from rural health clinics operated by the Dhaka Community Hospital Trust in the Sirajdikhan and Pabna Sadar Upazilas of Bangladesh. Between 2010 and 2013, health care workers invited families to participate in follow-up studies [19]. All children born during recruitment for the reproductive cohort study were eligible. Informed consent was provided by parents of all participants before enrollment. Reproductive health and follow-up studies were approved by the Human Research Committees at the Harvard T.H. Chan School of Public Health and Dhaka Community Hospital. Boston Children’s Hospital formally ceded review of the follow-up study to the Harvard T.H. Chan School. A total of 1613 women were enrolled in the reproductive health study, of which 1458 had a confirmed singleton pregnancy, were informed about the study, and provided written consent to participate. A total of 964 children participated in follow-up activities.

### Lead exposure

Suspected sources of Pb exposure in Mexico include traditional lead glazed pottery, alternative remedies or cosmetics, air pollutants, and nutritional sources [20]; the main source of Pb exposure in Bangladesh is suspected to be food contamination [21]. We collected venous whole blood from pregnant women participating in the Mexico cohort at delivery (± 12 h) using stainless steel phlebotomy needles and a single-stick lead-free vacutainer tube. We collected umbilical cord venous blood in trace element-free tubes at time of delivery from participants in the Bangladesh cohort. Samples were immediately frozen and kept at 4 °C during shipping to the Trace Metals Laboratory at Harvard T.H. Chan School of Public Health (Boston, MA). All samples were processed in a dedicated trace metal cleanroom outfitted with a Class 100 clean hood and using glassware that was cleaned by soaking in 10% HNO_3_ for 24 h and rinsed several times with 18Ω Milli-Q water. Blood samples were prepared for measurement of lead concentration by first weighing (~1 g) and then digesting samples in 2 mL concentrated HNO_3_ for 24 h. Samples were treated overnight with 30% hydrogen peroxide (1 mL per 1 g of blood) and then diluted to 10 mL with deionized water. Acid-digested samples were analyzed for total lead with a dynamic reaction cell inductively coupled plasma mass spectrometer (DRC-ICP-MS, Perkin Elmer, Norwalk, CT). The average of five replicate measurements for each individual sample was reported as final value. Recovery rates for lead in quality control and spiked samples were 92%–107%, and precision (percent relative standard deviation) was <10%. All measurements were greater than the average limit of detection, 0.2 μg/dL.

### Covariates

#### Mexico cohort

Study staff interviewed mothers at time of enrollment to obtain demographic data, including age, education (≥high school or <high school), parity, marital status, smoking during pregnancy, and exposure to environmental tobacco smoke. At time of birth, infant sex was recorded and infant birth weight, head circumference, and birth length were abstracted from hospital medical records. Gestational age at birth was estimated based on date of last menstrual period (LMP) and by the Capurro method [22]. LMP was used preferentially; estimates from the Capurro method were used only when the two methods differed by ±3 weeks. Study staff also recorded delivery type (vaginal, forceps, scheduled cesarean section, or emergency cesarean section). Interviewers administered the Home Observation for Measurement of the Environment (HOME) instrument when children were 1–24-months old to characterize the quality and extent of stimulation for children in their home environments [23].

#### Bangladesh cohort

At enrollment, we interviewed participating women to obtain information on age, education (>primary school or ≤primary school), parity, marital status, smoking history, and socioeconomic status. We assessed socioeconomic status by directly asking husbands their monthly income (<3000 taka, 3000–5000 taka, >5000 taka, or refused to respond). Gestational age at birth was determined by ultrasound measurement taken at time of enrollment, because few women could remember the date of their last menstrual period. Infant sex, birth weight, head circumference, and birth length were recorded at birth. Delivery type (vaginal, forceps, scheduled cesarean section, or emergency cesarean section) was also recorded. For the follow-up study, trained study staff administered questionnaires that collected medical histories and demographic information. Weight, height, and head circumference were measured. Maternal IQ was assessed using Raven’s Indices. Interviewers also administered the HOME instrument that had been previously translated and adapted for use in Bangladesh [23].

### Neurodevelopmental outcomes

In Mexico, child neurodevelopment was assessed at approximately 2 years of age using a Spanish version of the Bayley Scales of Infant and Toddler Development, Third Edition (BSID-III). In Bangladesh, we translated the BSID-III into Bengali and adapted it for use in rural Bangladesh (M.M.). Two primary outcomes (mental composite score and motor composite score) were derived by summing across raw scores of cognitive, language, and motor development for each participant: (1) mental development composite score (sum of cognition, expressive language, and receptive language); and (2) motor development composite score (sum of fine motor and gross motor). In both cohorts, trained study personnel who were unaware of participants’ blood Pb levels administered tests using standard protocols. An expert child neurologist (M.M.) and neuropsychologists (D.C.B., L.S.) oversaw administration of BSID-III, and quality control included frequent site visits and review of videotaped administration of neurodevelopmental assessments.

### Genotyping

Umbilical cord blood samples were collected at time of delivery from infants in both cohorts using an umbilical cord catheter and Paxgene DNA collection tubes (Qiagen, Venlo, Limberg). DNA was extracted from whole blood samples using Puregene DNA isolation kits (Gentra Systems, Minneapolis, MN) and stored at −20 °C. DNA samples were genotyped using the high density HumanOmni1-Quad BeadChip for the Mexico cohort and Illumina OmniExpressExome-8 BeadChip for the Bangladesh cohort (Illumina, San Diego, CA). Before analysis, a systematic quality evaluation was conducted on raw genotyping data according to the general quality control (QC) procedure described by Anderson [24]. Briefly, unqualified samples were excluded if they fit the following QC criteria: 1) overall genotype completion rates <95%; 2) gender discrepancies; 3) unexpected duplicates or probable relatives (based on pairwise identity by state value, PI_HAT in PLINK >0.185); 4) outlying heterozygosity rates >6 standard deviations from the mean; or 5) subjects identified as outliers using principal components analysis (PCA). Unqualified SNPs were excluded when they fit the following QC criteria: a) SNPs had a call rate < 95% in all samples; b) genotype distributions of SNPs deviated from those expected by Hardy-Weinberg equilibrium (*p* < 1.0 × 10^−6^); or c) SNPs were monomorphic in our study samples. After quality evaluation, a genotyping dataset of 552,487 SNPs were available for analysis. We also excluded very low birth weight infants (<1500 g), very premature births (<32 weeks gestation), and participants lacking birth outcomes data. The final sample sizes from the Mexico and Bangladesh cohorts for this analysis were 390 and 497, respectively (Table 1).

**Table 1:** Demographic characteristics of Bangladesh and Mexico cohorts

Characteristics	Mean (SD) or n (%)	
	Bangladesh (*n* = 497)	Mexico (*n* = 390)
Maternal education^a^		
High education	266 (53.5%)	231 (59.2%)
Low education	231 (46.5%)	159 (40.8%)
Maternal marital status at delivery		
Married or living with partner	497 (100%)	316 (81.0%)
Single, separated, or divorced	0 (0%)	74 (19.0%)
Prenatal exposure to second-hand smoke		
Smoker living in home	207 (41.6%)	140 (35.9%)
No smoker living in home	290 (58.4%)	250 (64.1%)
Child gender		
Male	241 (48.5%)	209 (53.6%)
Female	256 (51.5%)	181 (46.4%)
Delivery type		
Vaginal (including forceps)	303 (61.0%)	192 (49.2%)
Cesarean section	194 (39.0%)	198 (50.8%)
Maternal age (years)	22.8 (4.3)	27.7 (5.5)
Gestational age (weeks)	38.1 (1.7)	38.5 (1.6)
Birth weight (kg)	2.86 (0.40)	3.10 (0.43)
Head circumference (cm)	32.76 (1.43)	34.28 (1.48)
Cord blood Pb (μg/dL)	5.10 (6.51)	3.83 (2.70)
Bayley Scales of Infant Development, 24 months		
Mental composite raw score	112.7 (10.4)	108.6 (9.8)
Motor composite raw score	92.7 (5.0)	91.7 (4.5)

^a^Maternal education in Mexico cohort: < high school or ≥ high school. Maternal education in Bangladesh cohort: < primary school or ≥ primary school

### Mapping SNP to genes

The SNPs were mapped to genes in dbSNP database using GRCh37 build. Initially, for batch operation, we used stricter criteria with a narrow margin of 5 kb up- and down-stream of the RefSeq genes. For top-rank SNPs, we manually searched for all genes within a large 500 kb region flanking each SNP. However, this mapping approach was only used for exploring relevant biological scheme, but lack of the certainty of determining which gene(s) is affected by disease-associated SNPs.

### In vitro transcriptomic profiling for Pb toxicity

We previously performed next-generation RNA sequencing on hNSCs to determine the primary transcriptional response to Pb in these cells. hNSCs differentiated from the NIH-approved embryonic stem cell line H9 were grown in cell culture. Cells were exposed to 1 μM Pb and double-distilled water for 24 h before RNA harvesting. cDNA libraries were prepared using an Illumina RNA TruSeq v2 kit and sequenced on an Illumina High Seq 2000 for approximately 50 million reads per sample. Reads were aligned to the genome and gene count tables generated. Sequencing depth was normalized and differential expression called in edgeR implemented in Bioconductor (http://www.bioconductor.org/packages/release/bioc/html/edgeR.html) [25]. For all reported statistically significant results, read alignments were checked manually. Quantitative PCR (qPCR) using SYBR green was used to validate RNA sequencing results. The Pb concentration used in this study, 1 μM, is roughly twice the CDC level of concern for Pb in blood and reasonably estimates blood Pb concentrations observed in study populations [26, 27].

### Integrative analysis of GWIS and in vitro transcriptomic data

Incorporating biological functionality can boost the power of genetic mapping. We conducted in silico integrative evaluation of population-based genomic data and complementary in vitro transcriptomic data at gene and network levels to better understand the contribution of GxE interactions, particularly in identifying genes functionally relevant to metal-induced neurodevelopmental outcomes. Our hypothesis was that although candidate gene lists obtained from genomic and transcriptomic studies might or might not overlap at the gene level, there are sets of molecules that directly or indirectly interact with candidate genes at the network level.

We initially mapped a GWIS list of SNPs to a list of genes using SNP Annotation Tool (http://www.snp-nexus.org/). At the gene level, we matched GWIS gene lists and transcriptome gene lists to common identifiers defined by NCBI. From this we identified common gene(s) shared by GWIS and in vitro transcriptomic profiling. At the network level, we uploaded GWIS gene lists together with transcriptome gene lists into MetaCore™ GeneGo database for system biology (Thomson Reuters, New York, NY). Next, we used direct interaction algorithms to build gene network(s) consisting only of uploaded genes and their direct interactions, without adding other genes/objects from the GeneGo database. Finally, we identified and manually removed direct interactions between any pairs of genes either from GWIS or transcriptome lists.

### Statistical analysis

All statistical procedures were performed using R version 2.15.2 (https://www.r-project.org/) and PLINK v1.07 (http://zzz.bwh.harvard.edu/plink/). Distributional plots were examined for all variables, and univariate and multivariate regression analyses were conducted to estimate the effect of covariates on neurodevelopment. We log transformed Pb concentrations because the distribution was severely right-skewed with a few outliers.

To adjust for population stratification, we used EigenCorr, a PCA-based method that selects components based on their eigenvalues and their correlation with the phenotype [28]. Based on inspection of PCA plots and a review of literature, it was determined that the first two components would be sufficient to adjust for population stratification in our relatively homogenous samples.

Covariates were selected as potential confounders to include in models when associated with Pb and Bayley scores in bivariate models (*p* < 0.10) and using LASSO [29]. In both cohorts, the following covariates were included: child’s gender, child’s age at time of neurodevelopmental assessment, gestational age, parity, maternal education (binary), environmental tobacco smoke exposure (binary), and the first two principal components (calculated separately for each cohort). The final model was then: E(Y_i_) = β_0_ + β_1_*G_i_ + β_2_*E_i_ + β_3_*G_i_*E_i_ + γ*Z_i_, where Y_i_ is continuous neurodevelopment phenotype (mental or motor composite score), G_i_ is minor allele count for a given SNP, E_i_ is log concentration of Pb in maternal or umbilical cord blood, and Z_i_ is vector of additional covariates to adjust for confounding. We did not implement a discovery-replication analytic strategy, because these cohorts had different ethnic backgrounds, different exposure profiles, and the outcomes were assessed independently by teams in countries with different cultures. In addition to cohort-based individual analysis, meta-analyses were conducted by combining results across cohorts for both mental and motor composite scores. We used METAL to combine *p*-values for each GxE interaction term that was present in both Bangladesh and Mexico cohorts [30].

## Results

### Demographic characteristics

Demographic characteristics of participating mother–infant pairs from Bangladesh and Mexico cohorts are presented in Table 1. Compared to the Bangladesh cohort, mothers in the Mexico cohort were more likely to be single parents (*p* < 0.0001), to be older at time of delivery (*p* < 0.0001), and to deliver infants by Cesarean section (*p* = 0.0005). Cord blood Pb levels were significantly higher in the Bangladesh cohort (*p* < 0.0001). Mental composite scores at 2 years were correlated with motor composite scores in both cohorts (Bangladesh: *r* = 0.666, *p* < 0.0001; Mexico: *r* = 0.554, *p* < 0.0001). Raw mental and motor composite scores were significantly higher in the Bangladesh cohort than Mexico cohort (*p* < 0.0001), but these raw scores were not yet age-adjusted and differences likely reflect the older age of Bangladeshi children at neurodevelopmental assessment (mean age ± SD = 2.34 ± 0.23 years) compared to Mexican children (mean age ± SD = 2.04 ± 0.05 years). Given the differences in ethnic backgrounds in the two cohorts and that neurodevelopmental outcomes were assessed by different teams, raw scores were used in all analyses. All models were adjusted for age at time of assessment by including age as a covariate.

### GWIS of neurodevelopmental outcomes

We performed meta-analyses of genome-wide GxE interactions to identify common genetic variants associated with neurodevelopmental outcomes (mental and motor composite scores, individually) assessed at 2 years of age (Additional file 1: Figure S1). Although none of the 552,487 SNPs reached genome-wide significance in meta-analyses, we identified 20 top-ranked SNPs associated with mental composite score and 21 top-ranked SNPs associated with motor composite score, with *p* < 0.001 in both cohorts and *p*_*meta*_ < 5 × 10^−4^ (Table 2). We also identified 18 SNPs associated with mental and motor composite scores with *p*_*meta*_ < 1 × 10^−3^ in all analyses (Table 3). Many of these SNPs were mapped to genes with biological functions relevant to neurodevelopment (Additional file 2: Table S1). We further explored the functionalities of top-ranked SNPs by investigating Combined Annotation Dependent Deletion (CADD) scaled scores. CADD incorporates a variety of different annotations to predict the genomic substitutions having the most deleterious effects in humans [31]. Out of all GRCh37/hg19 reference SNVs (~8.6 billion), we identified 4 SNPs in the top 10% (CADD score > 10) of most deleterious substitutions in the entire human genome, including rs10015043 (intron variant of gene *PALLD*, CADD score = 13.68), rs17075573 (intron variant of gene *MYRIP*, CADD score = 13.66), rs3817591 (3′ UTR variant of gene *FMN1*, CADD score = 11.19), and rs2276102 (missense variant of gene *TMEM135*, CADD score = 11.02).

**Table 2:** Top SNPs demonstrating gene-environment interactions for mental and motor composite scores

		Position	Alleles	Mexico (*n* = 390)		Bangladesh (*n* = 497)		Meta-analysis	Direction^a^	
SNP	Chr	(bp)	Minor/Ref	*p*	MAF	*p*	MAF	*p*		Mapped genes
Mental composite scores										
rs9642758	8	35,721,159	A/G	0.0004	0.076	0.0023	0.123	4.35E-06	++	*UNC5D*
rs2276102	11	87,013,438	T/C	0.0028	0.411	0.0011	0.330	1.13E-05	++	*TMEM135*
rs7952312	11	99,789,888	T/G	0.0031	0.365	0.0019	0.440	2.05E-05	--	*CNTN5*
rs7556550	1	89,928,044	T/C	0.0038	0.382	0.0033	0.190	4.26E-05	--	*GBP5/GBP6/LRRC8B/LRRC8C*
rs619092	3	173,131,044	T/C	0.0018	0.248	0.0067	0.225	4.69E-05	--	*NLGN1*
rs10503970	8	34,866,368	A/G	0.0018	0.077	0.0073	0.116	4.98E-05	++	*UNC5D*
rs2020402	11	87,120,819	T/G	0.0017	0.343	0.0090	0.403	5.98E-05	--	*LOC101929137/ TMEM135*
rs2483678	1	10,582,047	T/C	0.0045	0.135	0.0043	0.308	6.31E-05	--	*PEX14*
rs2480786	1	10,567,658	A/G	0.0057	0.134	0.0037	0.299	6.94E-05	++	*PEX14*
rs10270302	7	150,213,019	T/C	0.0021	0.071	0.0095	0.079	7.53E-05	++	*GIMAP7*
rs17035156	1	10,585,527	A/C	0.0050	0.133	0.0048	0.302	7.84E-05	--	*PEX14*
rs4811754	20	55,392,174	A/C	0.0031	0.423	0.0079	0.245	8.68E-05	--	*TFAP2C*
rs1498706	12	93,996,980	A/G	0.0041	0.275	0.0066	0.161	9.04E-05	--	*MRPL42/SOCS2/CRADD*
rs10493836	1	90,574,778	T/C	0.0053	0.083	0.0067	0.068	0.00012	--	*ZNF326/LRRC8D*
rs10953862	7	79,063,644	A/G	0.0067	0.240	0.0060	0.292	0.00013	--	*MAGI2*
rs1335647	1	89,832,464	T/C	0.0093	0.438	0.0048	0.221	0.00014	++	*GBP6*
rs12120749	1	52,145,202	A/G	0.0098	0.253	0.0049	0.041	0.00015	--	*OSBPL9*
rs4734384	8	99,133,678	A/C	0.0075	0.260	0.0079	0.313	0.00019	--	*POP1*
rs1349784	11	99,806,211	T/G	0.0067	0.383	0.0098	0.409	0.00021	--	*CNTN5*
rs999995	10	78,735,498	A/G	0.0085	0.485	0.0098	0.419	0.00026	++	*KCNMA1*
Motor composite scores										
rs3817591	15	33,057,775	T/C	5.54E-05	0.427	0.0031	0.490	1.16E-06	--	*FMN1*
rs4308495	5	30,155,798	T/C	0.0016	0.089	0.0019	0.121	1.15E-05	++	
rs28694796	7	155,403,168	A/G	0.0006	0.081	0.0093	0.068	2.72E-05	--	*EN2/RBM33/CNPY1/SHH*
rs322172	17	25,659,288	A/G	0.0088	0.232	0.0010	0.263	2.97E-05	++	*MIR4522/WSB1/KSR1*
rs10015043	4	169,485,237	A/G	0.0040	0.422	0.0025	0.366	3.28E-05	++	*PALLD*
rs13353041	1	55,773,756	A/G	0.0080	0.051	0.0015	0.074	3.96E-05	++	*USP24/MIR442*
rs170602	20	56,019,162	T/C	0.0082	0.186	0.0016	0.210	4.19E-05	++	*SPO11/RAE1/RBM38/CTCFL/PCK1/ZBP1*
rs9945240	18	44,139,893	A/G	0.0067	0.062	0.0021	0.054	4.53E-05	++	*LOXHD1*
rs10022111	4	169,484,303	A/G	0.0018	0.459	0.0072	0.297	4.91E-05	--	*PALLD*
rs2318817	18	65,494,366	A/C	0.0025	0.048	0.0064	0.119	5.57E-05	++	*LOC643542*
rs2552531	2	218,698,323	A/G	0.0050	0.019	0.0038	0.066	6.19E-05	++	*TNS1*
rs17075573	3	40,131,246	T/C	0.0027	0.116	0.0086	0.082	8.38E-05	--	*MYRIP*
rs30732	5	66,151,551	T/C	0.0058	0.302	0.0052	0.123	9.42E-05	++	*MAST4*
rs6458566	6	47,361,788	A/G	0.0053	0.310	0.0056	0.467	9.50E-05	++	*TNFRSF21/CD2AP*
rs1647292	8	4,017,692	T/C	0.0031	0.158	0.0091	0.177	9.83E-05	--	*CSMD1*
rs1324800	13	48,388,487	A/G	0.0033	0.041	0.0089	0.078	0.00010	++	*SUCLA2*
rs16849242	3	162,188,734	T/C	0.0097	0.171	0.0049	0.333	0.00014	++	
rs17111847	1	55,749,478	T/C	0.0070	0.052	0.0070	0.067	0.00015	--	*USP24/MIR442*
rs11600036	11	81,001,092	A/G	0.0062	0.135	0.0080	0.433	0.00016	++	
rs10122848	9	25,368,388	T/G	0.0071	0.073	0.0077	0.229	0.00017	--	
rs12593647	15	87,117,255	A/C	0.0090	0.164	0.0090	0.262	0.00024	++	*AGBL1*

*SNP* Single-nucleotide polymorphism, *MAF* minor allele frequency

All models adjusted for child’s gender, child’s age at time of neurodevelopment testing, gestational age, parity, maternal education (binary), environmental tobacco smoke exposure (binary), and first two principal components of population stratification

^a^Effects of gene-environment interaction on the neurodevelopmental outcomes. “+”: increased risk; “-”: protective effect. The first “+/−” represents the effect observed in Mexico cohort, and the second “+/−” represents the effect observed in Bangladesh cohort

**Table 3:** Top SNPs demonstrating gene-environment interactions for both mental and motor composite scores

				MAF		Mental composite scores				Motor composite scores				
SNP	Chr	Alleles (Minor/Ref)	Position (bp)	Mexico	Bangladesh	*p*_Max_	*p*_Bang_	*p*_meta_	Dir^a^	*p*_Max_	*p*_Bang_	*p*_meta_	Dir^a^	Gene
rs12071731	1	T/G	6,276,167	0.047	0.126	0.0035	0.011	0.00013	++	0.020	0.012	0.00065	++	RNF207
rs2552531	2	A/G	218,698,323	0.019	0.066	0.0063	0.018	0.00037	++	0.0050	0.0038	6.19E-05	++	TNS1
rs645163	2	A/G	219,682,257	0.186	0.184	0.013	0.022	0.00083	--	0.0086	0.020	0.00054	--	PRKAG3/CYP27A1/ WNT6/TTLL4/WNT10A
rs755773	4	A/G	32,628,884	0.193	0.230	0.079	0.0034	0.00086	--	0.184	0.00079	0.00075	--	
rs11133230	4	A/G	32,640,520	0.280	0.336	0.101	0.00012	8.17E-05	--	0.139	9.87E-06	2.06E-05	--	
rs920908	5	T/C	41,288,697	0.128	0.350	0.197	0.00041	0.00052	--	0.0032	0.022	0.00026	--	MROH2B/LOC102723740/ C6/PLCXD3
rs32897	5	A/G	76,250,972	0.132	0.138	0.021	0.017	0.00099	--	0.014	0.020	0.00077	--	CRHBP
rs12201457	6	T/C	117,330,177	0.189	0.070	0.065	1.53E-05	9.71E-06	--	0.387	9.72E-05	0.00053	--	RFX6
rs3112346	7	A/G	135,387,495	0.270	0.268	0.032	0.0044	0.00042	--	0.069	0.0045	0.00092	--	SLC13A4
rs28694796	7	A/G	155,403,168	0.081	0.068	0.025	0.0028	0.00022	--	0.00063	0.0093	2.72E-05	--	EN2/RBM33/CNPY1/ SHH
rs10503970	8	A/G	34,866,368	0.077	0.116	0.0018	0.0073	4.98E-05	++	0.0019	0.031	0.00026	++	UNC5D
rs4269538	8	T/C	34,889,071	0.094	0.130	0.0019	0.055	0.00050	--	0.023	0.011	0.00068	--	UNC5D
rs7837686	8	T/C	142,674,928	0.441	0.449	0.040	0.0051	0.00058	++	0.013	0.018	0.00069	++	MROH5/MIR1302–7
rs6475430	9	T/C	20,430,468	0.250	0.338	0.050	0.00043	9.38E-05	--	0.0058	0.019	0.00035	--	MLLT3
rs10898112	11	A/G	69,934,085	0.243	0.102	0.038	0.0061	0.00066	--	0.233	0.00073	0.00099	--	ANO1
rs2168819	11	A/G	80,964,065	0.340	0.439	0.079	0.0014	0.00042	++	0.014	0.018	0.00070	++	
rs1950593	14	A/G	27,408,826	0.118	0.186	0.085	0.00087	0.00031	++	0.014	0.0077	0.00032	++	MIR4307
rs11848381	14	A/G	55,188,149	0.124	0.219	0.0071	0.041	0.00097	++	0.0010	0.031	0.00016	++	SAMD4A

*SNP* Single-nucleotide polymorphism *MAF* minor allele frequency

All models adjusted for child’s gender, child’s age at time of neurodevelopment testing, gestational age, parity, maternal education (binary), environmental tobacco smoke exposure (binary), and first two principal components of population stratification

^a^Effects of gene-environment interaction on the neurodevelopmental outcomes. “+”: increased risk; “-”: protective effect. The first “+/−” represents the effect observed in Mexico cohort, and the second “+/−” represents the effect observed in Bangladesh cohort

**Fig. 1:**
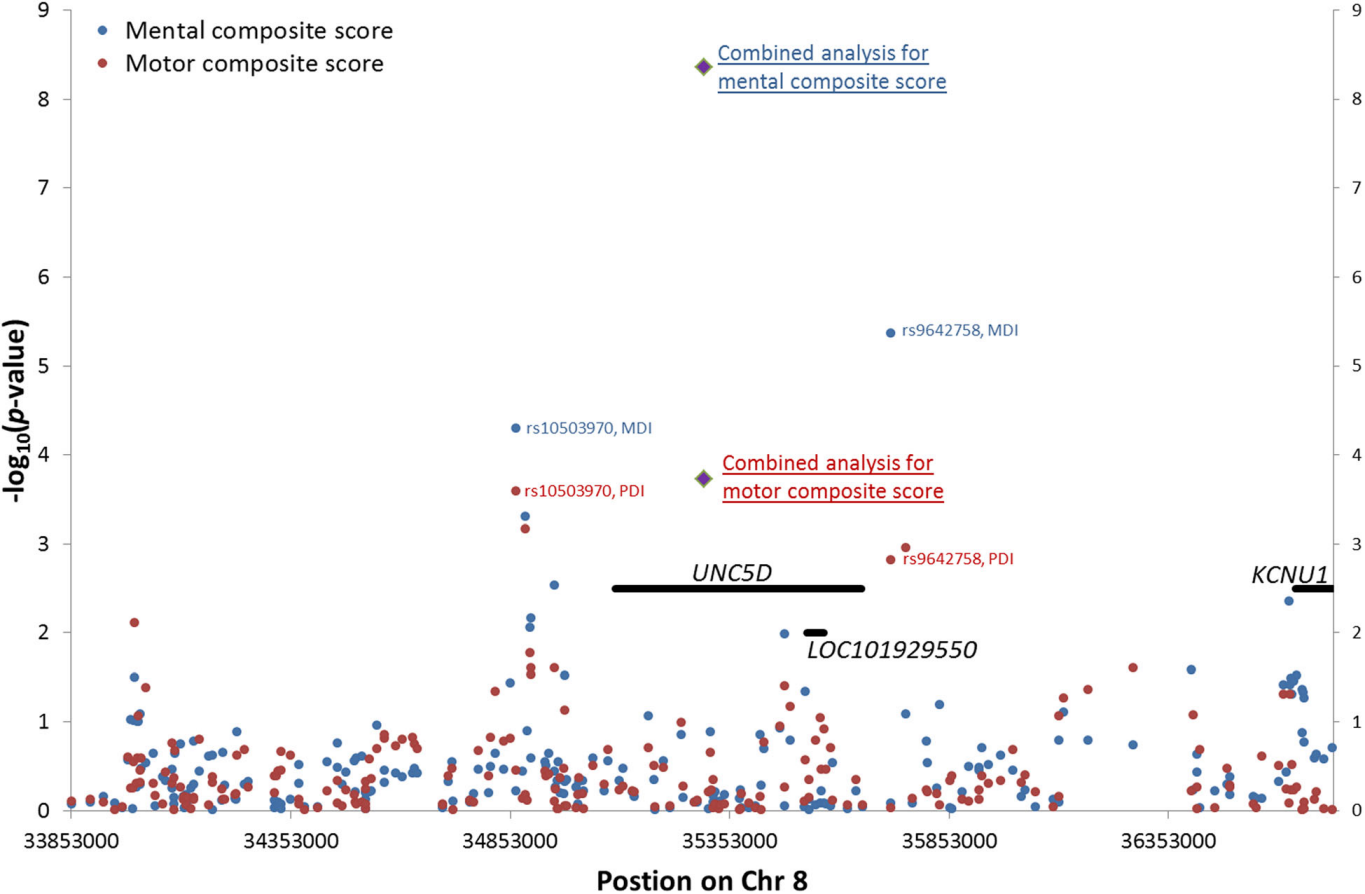
Regional association plot showing significant regions around *UNC5D* on chromosome 8. Results are reported for the most significant SNP, rs9642758, from genome-wide gene–environment effects analysis. *Blue circles* represent results of mental composite score analysis; *red circles* represent results of motor composite score. *Blue* and *red diamond* symbols represent combined analysis of SNP rs9642758 and rs10503970 for mental composite score and motor composite score, respectively

The top locus mapped to chromosome 8 and demonstrated strong association with mental composite score but moderate association with motor composite score (Table 3). For mental composite score, as shown in Fig. 1, the most strongly associated SNP (rs9642758) was located downstream of *UNC5D* (~87 kb), whereas the second most strongly associated SNP (rs10503970) was located upstream of *UNC5D* (~220 kb) (Additional file 3: Figure S2 and Additional file 4: Figure S3). *UNC5D* encodes a receptor for netrin that may be involved in axon guidance by mediating axon repulsion of neuronal growth cones in the developing nervous system [32].

Linkage disequilibrium analysis showed that the two SNPs were in linkage equilibrium (Bangladesh: D’ = 0.228, R^2^ = 0.001; Mexico: D’ = 0.108, R^2^ = 0.011) and had comparable main effects and GxE interactive effects on both mental and motor composite scores in each cohort (Table 4 **,** Additional file 5: Figure S4). We further used IMPUTE2 and the entire 1000 Genomes reference panel to impute the entire region between the two SNPs [33]. None of the imputed SNPs reached the significance level of either of the original SNPs (Additional file 6: Table S4). We next tested the joint effect of these two SNPs by creating a combined SNP, summing the number of minor alleles at rs9642758 and rs10503970. Compared with wild-type genotypes at both sites, the joint effect reached genome-wide significance for GxE interaction in models of mental composite score (*p* = 4.38 × 10^−9^), but not motor composite score (*p* = 1.87 × 10^−4^) (Table 4). We also conducted the analysis including both SNPs in the same model to evaluate if mild to moderate LD influenced results, but conclusions were unchanged (Additional file 7: Table S5).

**Table 4:** Joint effect of rs9642758 and rs10503970 locus on Chromosome 8^a^

	SNP_main_				Pb_main_				GxE Interaction			
Cohort	β	95% CI		*p*	β	95% CI		*p*	β	95% CI		*p*
Mental composite scores												
rs9642758 (minor allele: A; major allele as reference: G)												
Mexico	−8.16	−13.42	−2.90	0.0025	−1.93	−3.52	−0.34	0.0174	7.66	3.35	11.97	4.74E-04
Bangladesh	−3.41	−6.22	−0.60	0.0176	−0.44	−1.44	0.55	0.3821	2.93	0.91	4.95	0.0044
Meta												8.79E-06
rs10503970 (minor allele: A; major allele as reference: G)												
Mexico	−7.45	−13.42	−1.47	0.0147	−1.77	3.34	−0.19	0.0283	7.32	2.85	11.78	0.0013
Bangladesh	−3.52	−5.95	−1.08	0.0048	−0.49	−1.52	0.53	0.3452	2.36	0.69	4.04	0.0056
Meta												2.57E-05
rs9642758 & rs10503970												
Mexico	−6.13	−10.22	−2.05	0.0034	−2.33	−3.77	−0.88	0.0017	5.72	3.01	8.44	3.41E-05
Bangladesh	−3.82	−5.89	−1.75	3.20E-04	−1.23	−2.43	−0.03	0.0438	2.88	1.53	4.24	3.06E-05
Meta												4.38E-09
Motor composite scores												
rs9642758 (minor allele: A; major allele as reference: G)												
Mexico	−2.62	−5.17	−0.08	0.0435	−0.21	−0.98	0.56	0.5948	2.82	0.31	5.33	0.0271
Bangladesh	−1.13	−2.53	0.28	0.1153	0.48	−0.02	0.97	0.0597	0.90	−0.04	1.83	0.0587
Meta												0.0041
rs10503970 (minor allele: A; major allele as reference: G)												
Mexico	−4.46	−7.35	−1.57	0.0026	−0.22	−0.98	0.54	0.5630	3.51	0.90	6.11	0.0081
Bangladesh	−1.48	−2.69	−0.27	0.0166	0.39	−0.12	0.91	0.1306	0.94	−0.10	1.98	0.0754
Meta												0.0021
rs9642758 & rs10503970												
Mexico	−2.68	−4.84	−0.52	0.0154	−0.42	−1.13	0.29	0.2428	2.36	0.51	4.22	0.0121
Bangladesh	−1.52	−2.62	−0.42	0.0071	0.16	−0.38	0.69	0.5673	1.04	0.30	1.78	0.0056
Meta												1.87E-04

*SNP* Single-nucleotide polymorphism, *MAF* minor allele frequency

^a^Model: Y = β_0_ + β_1_*G + β_2_*E(Pb) + β_3_*G*E + γ*Z, adjusted for child’s gender, child’s age at time of neurodevelopment testing, gestational age, parity, maternal education (binary), environmental tobacco smoke exposure (binary), and first two principal components of population stratification

### Gene expression trajectories of top-mapped genes

We examined each top-mapped gene for association with previously defined co-expression networks of a spatiotemporal transcriptome of the human brain and retrieved temporal trajectories of neurodevelopment from the Human Brain Transcriptome public database (http://hbatlas.org/) [34]. Twenty-two of 67 mapped genes were identified in five co-expression networks (Additional file 2: Table S1). The two largest modules, M2 and M20, appeared to be simultaneously co-expressed across all brain regions with opposite developmental trajectories (Additional file 8: Figure S5). The M2 module, containing eight mapped genes, was associated with a progressive increase in gene expression across all brain regions, starting at the embryonic period and remaining at a high level of expression after birth. The M20 module, containing seven mapped genes (including *UNC5D*), was associated with a progressive decrease in gene expression across all brain regions starting from the embryonic period. A third module, M15, contained three mapped genes and demonstrated a similar neurodevelopmental trajectory as the M2 module, but only in certain regions: the neocortex, hippocampus, amygdala, and striatum.

### Integrative analysis of GWIS and in vitro transcriptomic data

To retrieve potential profiles of biological functions associated with genes mapped to top-ranked SNPs, we performed integrative analysis with in vitro transcriptomic data. We previously performed next-generation sequencing of transcripts (RNAseq) in hNSC-derived neurons that were exposed to Pb and identified both known and novel RNA transcripts that are responsive to Pb exposure. This transcriptomic analysis identified that Pb-induced expression was enriched for genes related to oxidative stress and endoplasmic reticulum (ER) stress [35].

Less stringent criteria for GWIS data (*p* < 0.05 in both cohorts; *p*_*meta*_ < 5 × 10^−3^) were used to identify 492 SNPs for mental composite score and 578 SNPs for motor composite score, mapped to 210 and 248 genes, respectively. By comparing top lists of genes from our GxE analyses and the list of in vitro transcriptomic analyses, we identified one common gene, *SLC1A5,* with an intron variant (rs1644343: mental composite score *p*_*Mexico*_ = 0.026; *p*_*Bangladesh*_ = 0.037; and *p*_*meta*_ = 0.0026) that progressively decreased gene expression across all brain regions from embryonic to postnatal periods, with resulting low-level expression after birth (Additional file 8: Figure S5). *SLC1A5* encodes an integral membrane transport protein for a neurotransmitter and is potentially involved in synaptic function, neuronal development, and excitotoxicity [36]. In hNSCs, Pb exposure significantly increased expression of *SLC1A5* (fold change = 1.3; *p* = 2.19 × 10^−10^; FDR = 2.55 × 10^−7^).

**Fig. 2:**
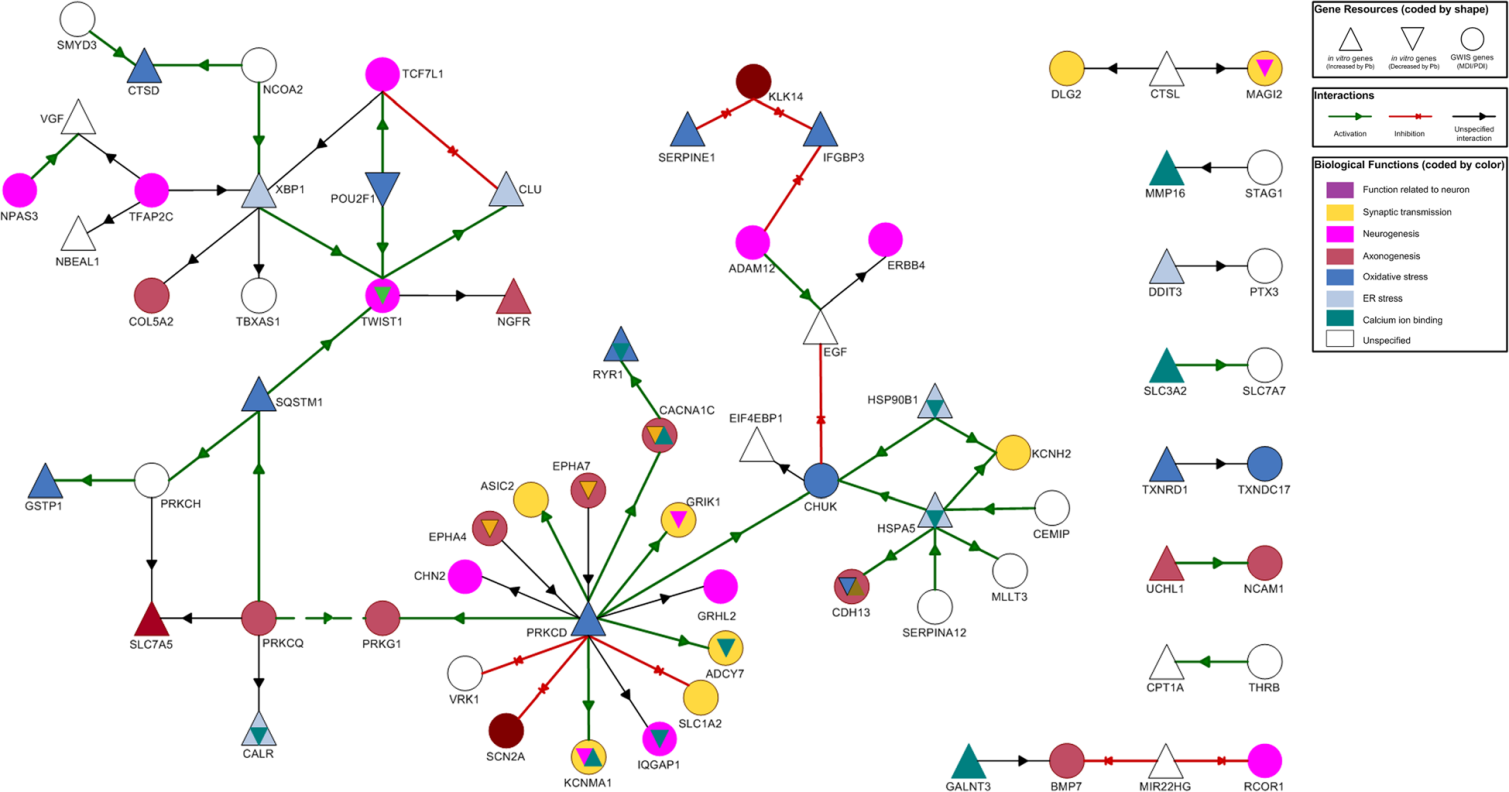
Network of directional interactions between GWIS genes and in vitro transcriptomic genes. *Round* symbols represent genes identified by GWIS. *Triangle* symbols represent genes selected by in vitro transcriptomic assays, with upward *triangles* indicating Pb-induced genes and downward *triangles* indicating Pb-suppressed genes. Biological functions were annotated by NCBI, UCSC Genome Bioinformatics, and literature review

We investigated further the direct gene-gene interactions between top genes from GxE interaction analyses and top genes from in vitro assays. We identified 63 interactions between 44 GxE genes and 28 in vitro genes (Additional file 9: Table S2). Gene Ontology enrichment analysis showed that expression of GxE genes was highly enriched in brain tissue [GO:0043005, neuron projection, *p* = 2.61 × 10^−4^; GO:0014069, postsynaptic density, *p* = 6.11 × 10^−3^ (with Bonferroni correction)]. Genes’ biological functions were also enriched for nervous system development (GO:0007399, *p* = 1.30 × 10^−9^ with Bonferroni correction). The majority of these interactions were interconnected, forming a large network (Fig. 2). Network hub genes (≥5 connections) included two genes (*CHUK* and *TWIST1*) from GxE interactions and three genes (*PRKCD*, *HSPA5*, and *XBP1*) from in vitro assays. Interactions included 33 activations (via mechanisms of 13 bindings, 1 cleavage, 1 deubiquitination, 8 phosphorylations, and 10 transcription regulations), 11 inhibitions (via mechanisms of 2 bindings, 2 cleavages, 5 miRNA bindings, and 2 transcription regulations), and 19 unspecified interactions. Thirty-four interactions (54%) were between in vitro genes and GxE genes.

## Discussion

We present the first multi-ethnic genome-wide study to discover gene–Pb exposure interactions associated with neurodevelopmental outcomes. Although there are distinct ethnic and cultural backgrounds, socioeconomic conditions, and Pb exposure profiles in the two cohorts examined, we identified one locus on chromosome 8 reaching genome-wide significance for joint effects of two independent SNPs (close to linkage equilibrium) on mental composite scores. Within each cohort, the two SNPs had similar main effects and GxE interactive effects, and coefficients for Pb were also similar in magnitude. Results of joint effects of the two SNPs were consistent with effects of individual SNPs and had markedly increased statistical significance. SNPs rs10503970 and rs9642758 are separated by about 855 kb, comprising the entire genomic region of *UNC5D*, from ~227 kb upstream to ~67 kb downstream, respectively. Moreover, both SNPs were in either strong or moderate linkage disequilibrium (LD) with certain SNPs within *UNC5D* which might have potential impacts via LD-linked SNPs (Additional file 3: Figure S2). However, there were no known eQTL or functions related to these LD-linked SNPs. Further, this locus contains few genes besides *UNC5D*, with the closest one (*KCNU1*) located over 900 kb downstream of SNP rs9642758. Thus, *UNC5D* appears to be the candidate gene of this locus for GxE interaction on mental development. Similarly, in a study of prostate cancer, despite a weak association between prostate cancer risk and a missense change in *HSD3B1* genes, the joint effects of two polymorphisms in the *HSD3B* gene family demonstrate stronger evidence of association [37].

*UNC5D* encodes a member of the human dependence receptor UNC5 family for netrin. Netrins control guidance of CNS commissural axons and peripheral motor axons via association with either DCC (DCC netrin 1 receptor) or some UNC5 receptors, leading to axon attraction or repulsion, respectively [38]. UNC5D is specifically expressed in layer 4 of primary sensory areas of the developing neocortex, and thus may be involved in axon guidance by mediating axon repulsion of neuronal growth cones during neurodevelopment [32]. Temporal trajectory profiling from the Human Brain Transcriptome project suggests that expression of *UNC5D* peaks during mid to late pregnancy, gradually decreases, and is then maintained consistently at a relatively low level after birth (Additional file 8: Figure S5). UNC5D also acts as a dependence receptor required for apoptosis induction when not associated with netrin [39].

A chromosomal breakpoint of apparently balanced chromosome rearrangements was identified in intron 5 of *UNC5D* in a family with neurodevelopmental disorders [40]. The proband was noted with a developmental delay at 18 months of age affecting walking and speech, as well as with comprehension and behavioral difficulties at 9 years of age. A younger sister with the same translocation had language delay as well as low-to-average fine and gross motor skills at 2 years of age.

In our GxE interaction models, the main effects for Pb levels in prenatal blood were associated with lower mental composite scores in Mexico cohort, but not in the Bangladesh cohort. Pb is a well-known neurotoxicant; nonetheless, some studies have not observed significant adverse associations [41]. A prospective study revealed that fetal lead exposure was associated with the Bayley Mental Developmental Index (MDI) at 3 and 6 months, but no statistically significant relationships were observed with Bayley MDI scores at 3 years of age [42]. There may be several plausible explanations for a lack of a main Pb effect in the 2–4 year old Bangladeshi children, including an exposure range that is too narrow, or co-exposures to other neurotoxicants that may overwhelm a possible lead effect. However, the direction of residual and/or unmeasured confounding by co-exposures could be difficult to predict given the complex correlations between neurotoxicants in different areas of Bangladesh.

We found that minor alleles of SNPs rs10503970 and rs9642758 were significantly associated with lower neurodevelopment scores. However, the coefficient of GxE interaction was positive, which is in the opposite direction of SNPs and Pb exposure (Table 4). We expected to observe a synergy between risk factors resulting in even lower neurodevelopment scores, but our findings contradict this expectation. A direct interpretation of these findings is that, in contrast to the adverse effects of Pb exposure in the presence of wildtype genotypes, Pb exposure counteracts the detrimental effects of minor alleles on mental and motor composite scores at these sites. A possible explanation is that minor alleles may directly or indirectly change the spatial–temporal trajectories of *UNC5D* expression in the brain, resulting in deleterious effects on neurodevelopment. Pb neurotoxicity can arise by targeting multiple sites at multiple levels—molecular, cellular, inter-cellular, and tissue—which may also perturb spatial–temporal trajectories of *UNC5D* and offset detrimental effects of minor alleles.

In addition, there was a significant interaction between Pb levels and two SNPs on mental development scores in two genetically different populations and with different distributions of genotypes, strongly suggesting that *UNC5D* is an important candidate within the biological pathways of Pb neurotoxicity. However, our genome-wide study could not provide further evidence, so future functional research should focus on investigating and comparing the effects of these SNPs on biological functions of *UNC5D*, especially on spatial–temporal trajectories of brain tissue. Additionally, future research should also examine functional changes of *UNC5D* induced by Pb exposure.

By cross-evaluation of top gene lists between genome-wide GxE analyses and in vitro global expression profiling induced by Pb treatment, we identified one common gene, *SLC1A5*, that ranked high (15th) in in vitro transcriptomic assays but ranked relatively low (368th) in GxE analyses for mental composite score (intronic SNP rs1644343). *SLC1A5,* a member of the solute carrier 1 (*SLC1*) family, encodes a plasma membrane transporter for the neurotransmitter glutamate [43]. Major functions of glutamate transporters in the brain, expressed in both neurons and astrocytes, are removal of released glutamate from the synaptic cleft and initiation of the recycling cascade for restoring released glutamate in synaptic vesicles. Thus, glutamate transporter malfunction has been implicated in various nervous system diseases [44–46].

Pb is a ubiquitous environmental heavy metal pollutant whose toxic effects are principally manifested in the CNS of immature organisms [47]. Even at low doses, Pb is a potent neurodevelopmental toxicant associated with cognitive deficits and related behavioral disturbances [48–52]. The molecular basis of observed changes include selective blockade of glutamatergic synapses with a subsequent decrease in long-term potentiation, which underlies learning and memory processes [53, 54]. A possible mechanism is that all glutamate transporters possess specific cysteine residues with redox-active-SH groups, which regulate glutamate transport via the s-glutathionylation process, and these cysteine residues are vulnerable to biological and chemical oxidants, such as heavy metals. Reduced transporter function leads to neurotoxic levels of extracellular glutamate, resulting in neuronal damage via oxidative and excitotoxic mechanisms, which often operate together [55]. Beyond effects at the protein level, our *SLC1A5* results provide evidence that Pb toxicity also has effects at transcriptomic and genomic levels on glutamate transporters.

This study incorporated data from cohorts with different experimental designs and different biomarkers of prenatal Pb exposure. It is therefore not surprising that few common genes (only *SLC1A5*) could be identified from top-ranked GWAS gene lists and from transcriptomic studies (in vitro RNAseq), because there are subsets of genes from different cohorts that might directly or indirectly interact with each other at the network level under a general biological scheme of Pb neurotoxicity. Using stringent criteria of direct gene–gene interactions between studies with exclusion of interactions within studies, a large 52-gene network of direct gene–gene interactions revealed from integrative analysis supported our hypothesis (Fig. 2). This network had five hub genes (≥5 direct interactions), including two genes from GWIS (*CHUK* and *TWIST1*) and three genes from in vitro assays (*PRKCD*, *HSPA5*, and *XBP1*). There were direct interactions among hub genes, further dividing them into two subgroups, *XBP1–TWIST1* (21 genes) and *PRKCD*–*CHUK*–*HSPA5* (31 genes), connected by an interaction between two GWIS-identified genes, *PRKCQ* and *PRKG1*.

We found that the network contained three members of the protein kinase C family—*PRKCD* (PKCδ), *PRKCH* (PKCη), and *PRKCQ* (PKCθ)—that are known signal transducers in the CNS, with roles regulating synaptic development and signaling involved in learning and memory [54]. PKC is well established as one of the major molecular targets of Pb toxicity, and the effect of Pb on PKCs depends on Pb concentrations and periods of exposure in different regions of the brain. As a biological and physiological analogue to Ca^2+^, Pb can directly activate Ca^2+^-dependent PKCs (α, β1, β2, and γ) at ~1000 times less than the concentration of required calcium [56]. At higher concentrations estimated to exist in the brain tissue of environmentally exposed humans (μM range), Pb inhibits kinase activity of all PKCs in in vitro assays [57, 58]. Interestingly, all three PKCs identified from the network belonged to a PKC subset called novel PKCs (δ, ε, η, and θ), which are Ca^2+^-independent. Among them, *PRKCD* was a hub gene with the highest density of connections in the network (15 direct interactions with GWIS-identified genes), and *PRKCD* expression was significantly increased by Pb treatment (fold change = 1.4; *p* = 4.0 × 10^−4^; FDR = 0.055). PKCδ is actively involved in regulation of cell apoptosis, especially through a mitochondrial dysfunction pathway, in response to a large and diverse array of apoptotic stimuli [59]. Oxidative stress, often induced by metal ion exposure, results in overexpression and activation of PKCδ, and activated PKCδ translocates to mitochondria and alters mitochondrial functions, such as decreased membrane potential and release of cytochrome C [60].

Besides *PRKCD*, the other two hub genes identified from in vitro assays, *HSPA5* and *XBP1*, were key genes in ER stress pathways, a major cellular target of oxidative stress manifested at the maternal–fetal interface from early pregnancy onwards [61]. Growing evidence indicates that some of the toxic effects of Pb are oxidative stress via both ER stress and mitochondrial dysfunction [62]. In vitro studies reveal that Pb exposure upregulates gene and protein expression of HSPA5 (GRP78), a molecular chaperone that binds transiently to proteins traversing through the ER and facilitates their folding, assembly, and transport [63]. Consistently, we observed increased expression of *HSPA5* in in vitro assays (fold change = 1.2; *p* = 1.62 × 10^−6^; FDR = 7.18 × 10^−4^). Moreover, Pb directly binds to HSPA5, and Pb-bound HSPA5 stimulates the unfolded protein response of ER stress, which can also be activated by XBP1 [63]. Further, among the remaining 16 network genes identified from in vitro assays, 10 genes were involved in oxidative stress (e.g., *CALR*, *CLU,* and *HSP90B1,* which encode chaperones involved in ER stress) as revealed by literature review and functional annotations at UCSC Genome Bioinformatics (http://genome.ucsc.edu/).

Although evidence suggests that two hub genes identified by GWIS participate in modulation of oxidative stress via the NF-κB pathway [64], both *CHUK* and *TWIST1* play important roles in early neurogenesis by governing cell fate decisions of neural differentiation at the neural crest stage [65, 66]. In contrast to the majority of genes from in vitro assays involved in processes relevant to oxidative stress, many GWIS-identified genes within the network had functions related to neurodevelopment, such as axonogenesis, neurogenesis, and synaptic transmission (Additional file 10: Table S3). Specifically, *SLC1A2,* also a member of the *SLC1* family*,* encodes another plasma membrane transporter for the neurotransmitter glutamate, and *GIRK2* encodes an N-methyl-d-aspartate type of excitatory amino acid receptor. All of these genes are involved in glutamatergic synapses, which are direct targets for Pb’s effects in the brain [54]. Taken together, through a common theme of Pb exposure, enrichment of oxidative stress genes (mainly from in vitro assays) and neurodevelopmental genes (mainly from GWIS) within the large network of integrative analysis suggests that certain genetic polymorphisms within/near genes relevant to neurophysiological functions might modify Pb exposure effects via oxidative stress on neurodevelopmental outcomes.

The major limitation of this study was that Pb exposure was only assessed from blood collected at birth in both cohorts. Several GxE interactions, such as *UNC5D* and *SCL1A5*, also play important roles in neurodevelopment during early- or mid-stage pregnancy. On the other hand, neurodevelopment was assessed at 2 years of age, and postnatal Pb exposure, which also is a time critical for neurodevelopment, was not measured [67]. Lack of complete information on timing and duration of Pb exposure might further reduce the power of detecting GxE interactions within two different ethnic groups with relatively small sample sizes. Moreover, study cohorts had significantly different profiles of metal exposure. The Bangladesh population is well known for high-level arsenic exposure in addition to Pb exposure [68]. In a Mexican cohort similar to the present study, we previously found that high manganese co-exposure increased Pb toxicity among young children [49]. Despite the importance of examining joint exposures to toxicants, most previous toxicological studies have focused on a single agent and either do not measure or do not adjust for potential confounders or modifying effects of other chemicals. For the purpose of meta-analysis across cohorts, we also only focused on Pb exposure because it was commonly measured in both cohorts.

## Conclusion

In conclusion, GWIS revealed several candidates containing genetic polymorphisms that modified the response to prenatal Pb exposure on neurodevelopmental outcomes. Although their biological functions were generally related to neurodevelopment, these candidates seemed to play different roles at different developmental stages. Follow-up integrative analysis suggested that some of the candidates might have functions via Pb-induced oxidative stress. Our findings highlight the importance of exposure timing that corresponds to a critical developmental window in GxE interaction research. Future research should emphasize comprehensive measurement of exposure at various developmental stages.

## Additional files

Additional file 1: Figure S1. Q-Q plots of GWIS. (DOCX 9 mb)

Additional file 2: Table S1. Functional annotation of GWIS genes. (XLSX 19 kb)

Additional file 3: Figure S2. LD structures of chromosome 8 locus containing gene *UNC5D*. (DOCX 3 mb)

Additional file 4: Figure S3. The stratified scatterplots show how the effect of Pb on Mental and Motor Composite Score is modified by the number of minor alleles at the two top SNPs. In particular, we can see how both scores appear to fall as Pb increases for subjects with no minor alleles (black symbols), but scores appear to rise as Pb increases for subjects with one minor alleles (blue symbols). This trend occurs for both SNPs and both outcomes, showing the significant interaction effect between the two SNPs and Pb concentration. (DOCX 169 kb)

Additional file 5: Figure S4. Manhattan plots displaying genetic effects from genome-wide association of meta-analysis on mental or motor composite scores. (DOCX 5 mb)

Additional file 6: Table S4. Result of Imputed SNPs between rs9642758 and rs10503970. (XLSX 170 kb)

Additional file 7: Table S5. Analysis Gene-environment Interactions with Two SNPs. (XLSX 9 kb)

Additional file 8: Figure S5. Spatial and temporal expression trajectories of top genes associated with neurodevelopmental outcomes. (DOCX 1 mb)

Additional file 9: Table S2. Integrative analysis of direct gene-gene interactions between GWIS genes and in vitro transcriptomic assay genes. (XLSX 18 kb)

Additional file 10: Table S3. Functional annotation of genes from integrative analysis. (XLSX 49 kb)

## Abbreviations

BSID-III: Bayley Scales of Infant and Toddler Development, Third Edition
CNS: Central nervous system
ER: Endoplasmic reticulum
GWAS: Genome-wide association study
GWIS: Genome-wide gene-environment interaction study
GxE: Gene–environment
hNSC: Human neural stem cell
HOME: Home Observation for Measurement of the Environment
LD: Linkage disequilibrium
Pb: Lead
PCA: Principal components analysis
QC: Quality control
qPCR: Quantitative PCR
RNAseq: Next-generation sequencing of transcripts
SNP: Single nucleotide polymorphism
