# Supplementary figures and images for: Genome-wide gene by lead exposure interaction analysis identifies UNC5D as a candidate gene for neurodevelopment
Source: Environ Health. 2017 Jul 28;16:81. doi: 10.1186/s12940-017-0288-3 (PMC5534076; doi:10.1186/s12940-017-0288-3)

**Supplementary Figure S1. Q-Q plots of GWIS**


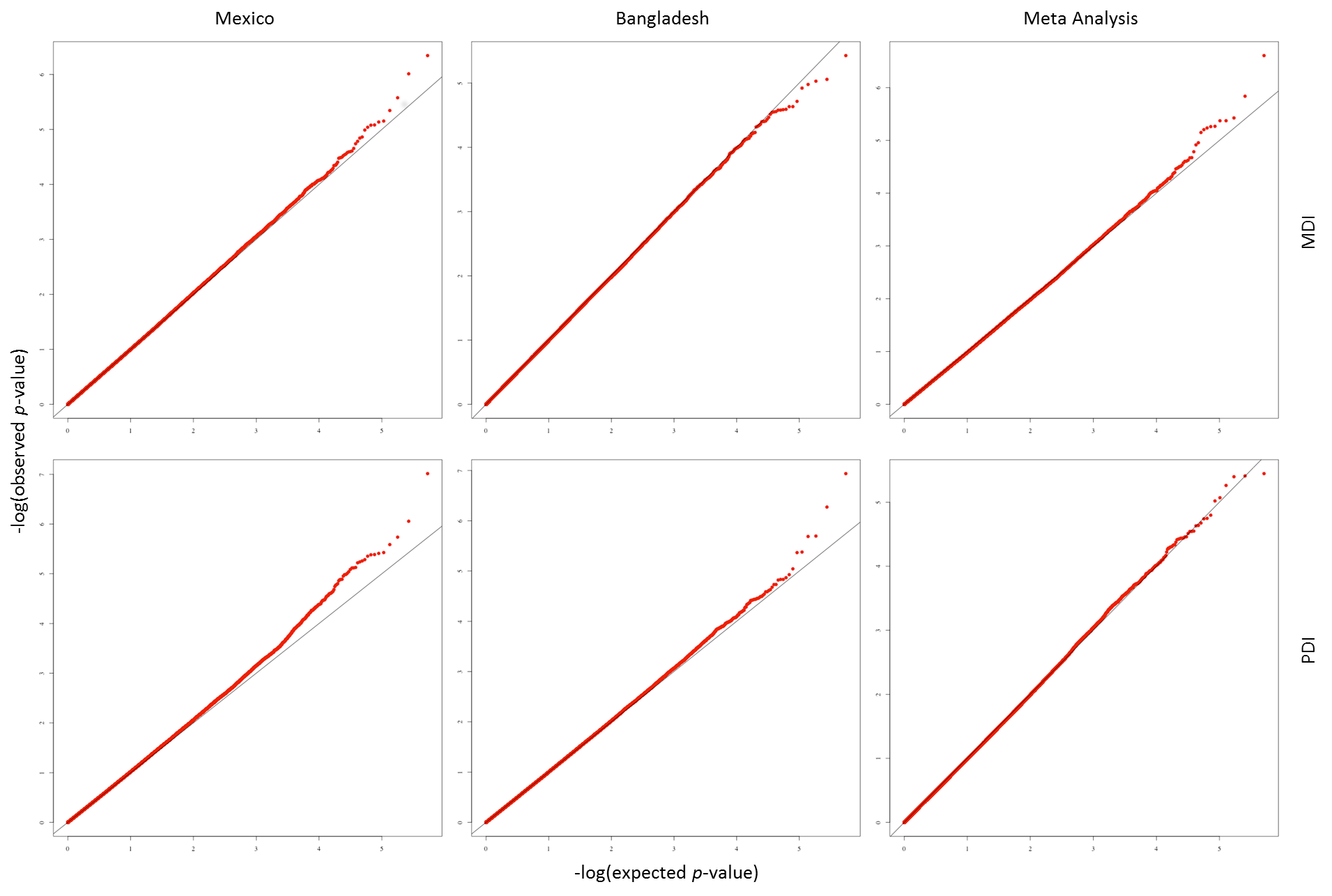

Supplement: Supplementary file 1 — Q-Q plots of GWIS. (DOCX 9 mb) [file 12940_2017_288_MOESM1_ESM.docx]

**Supplementary Figure S2. LD structures of chromosome 8 locus containing gene *UNC5D***


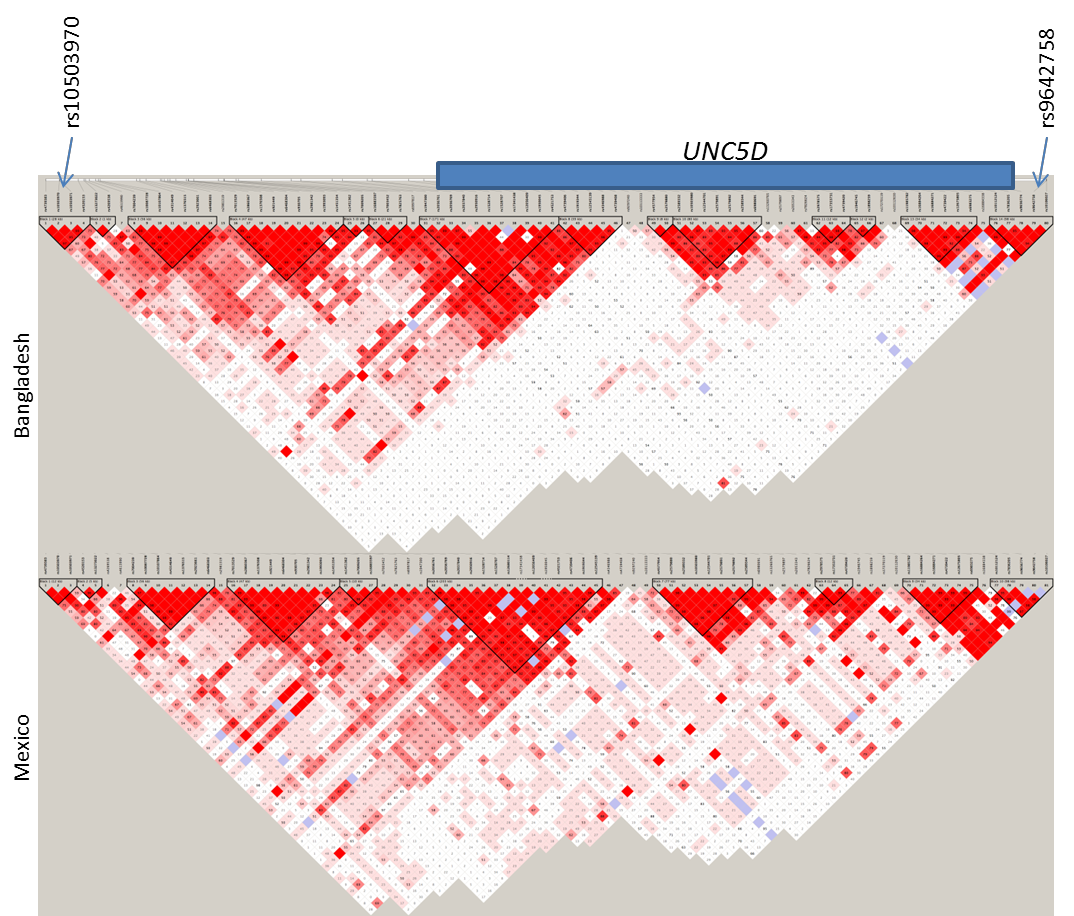

Supplement: Supplementary file 3 — LD structures of chromosome 8 locus containing gene UNC5D. (DOCX 3 mb) [file 12940_2017_288_MOESM3_ESM.docx]
