# Supplementary material for: Genome-wide gene by lead exposure interaction analysis identifies UNC5D as a candidate gene for neurodevelopment
Source: Environ Health. 2017 Jul 28;16:81. doi: 10.1186/s12940-017-0288-3 (PMC5534076; doi:10.1186/s12940-017-0288-3)
Supplement: Supplementary file 4 — The stratified scatterplots show how the effect of Pb on Mental and Motor Composite Score is modified by the number of minor alleles at the two top SNPs. In particular, we can see how both scores appear to fall as Pb increases for subjects with no minor alleles (black symbols), but scores appear to rise as Pb increases for subjects with one minor alleles (blue symbols). This trend occurs for both SNPs and both outcomes, showing the significant interaction effect between the two SNPs and Pb concentration. (DOCX 169 kb) [file 12940_2017_288_MOESM4_ESM.docx]

**Supplementary Figure S3. The stratified scatterplots show how the effect of Pb on Mental and Motor Composite Score is modified by the number of minor alleles at the two top SNPs. In particular, we can see how both scores appear to fall as Pb increases for subjects with no minor alleles (black symbols), but scores appear to rise as Pb increases for subjects with one minor alleles (blue symbols). This trend occurs for both SNPs and both outcomes, showing the significant interaction effect between the two SNPs and Pb concentration.**

**
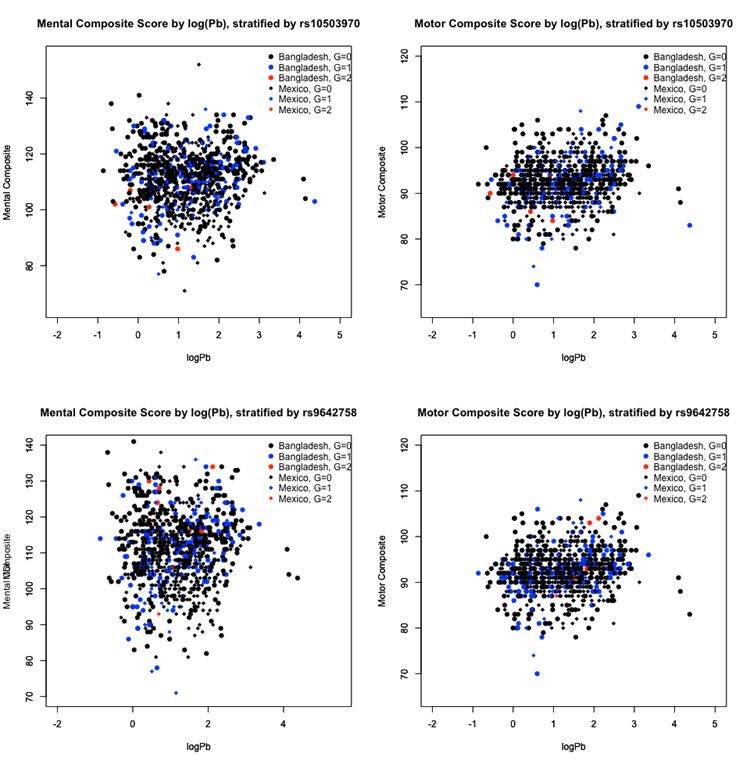
**
