# Supplementary material for: Genome-wide gene by lead exposure interaction analysis identifies UNC5D as a candidate gene for neurodevelopment
Source: Environ Health. 2017 Jul 28;16:81. doi: 10.1186/s12940-017-0288-3 (PMC5534076; doi:10.1186/s12940-017-0288-3)

**Supplementary Figure S4. Manhattan plots displaying genetic effects from genome-wide association of meta-analysis on mental or motor composite scores**


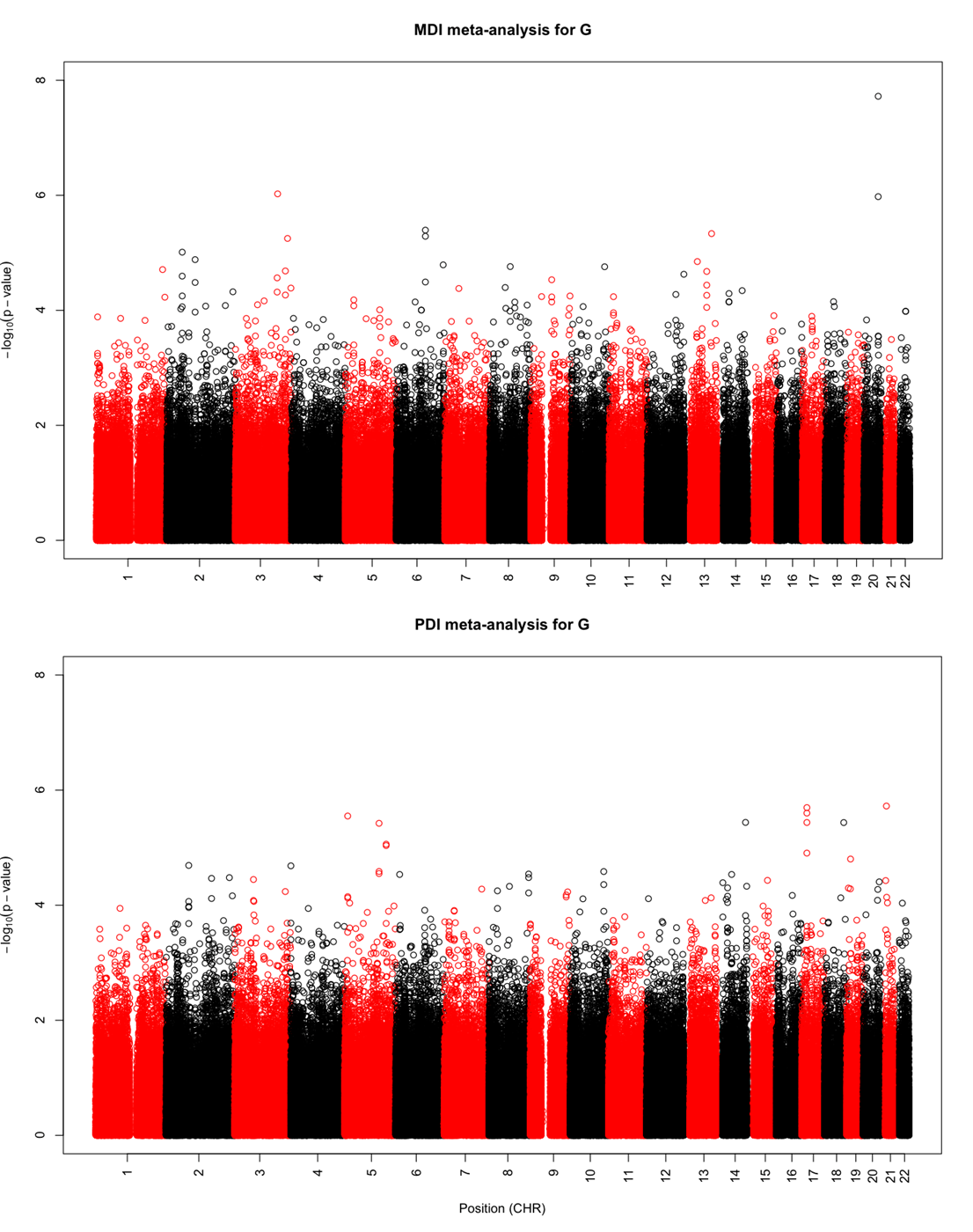

Supplement: Supplementary file 5 — Manhattan plots displaying genetic effects from genome-wide association of meta-analysis on mental or motor composite scores. (DOCX 5 mb) [file 12940_2017_288_MOESM5_ESM.docx]
