# Supplementary material for: Genome-wide gene by lead exposure interaction analysis identifies UNC5D as a candidate gene for neurodevelopment
Source: Environ Health. 2017 Jul 28;16:81. doi: 10.1186/s12940-017-0288-3 (PMC5534076; doi:10.1186/s12940-017-0288-3)
Supplement: Supplementary file 8 — Spatial and temporal expression trajectories of top genes associated with neurodevelopmental outcomes. (DOCX 1 mb) [file 12940_2017_288_MOESM8_ESM.docx]

**Supplementary Figure S5. Spatial and temporal expression trajectories of top genes associated with neurodevelopmental outcomes.**


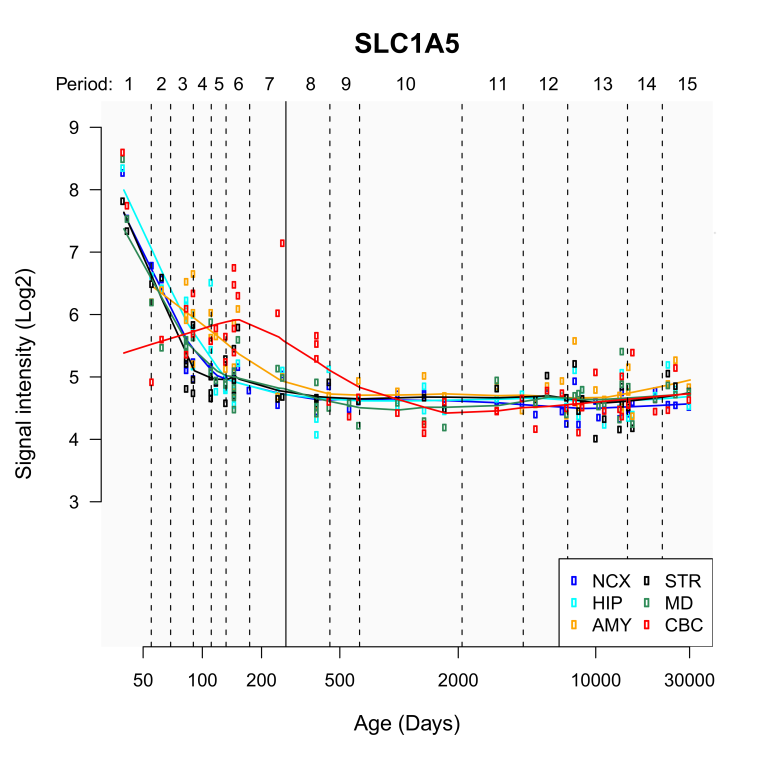

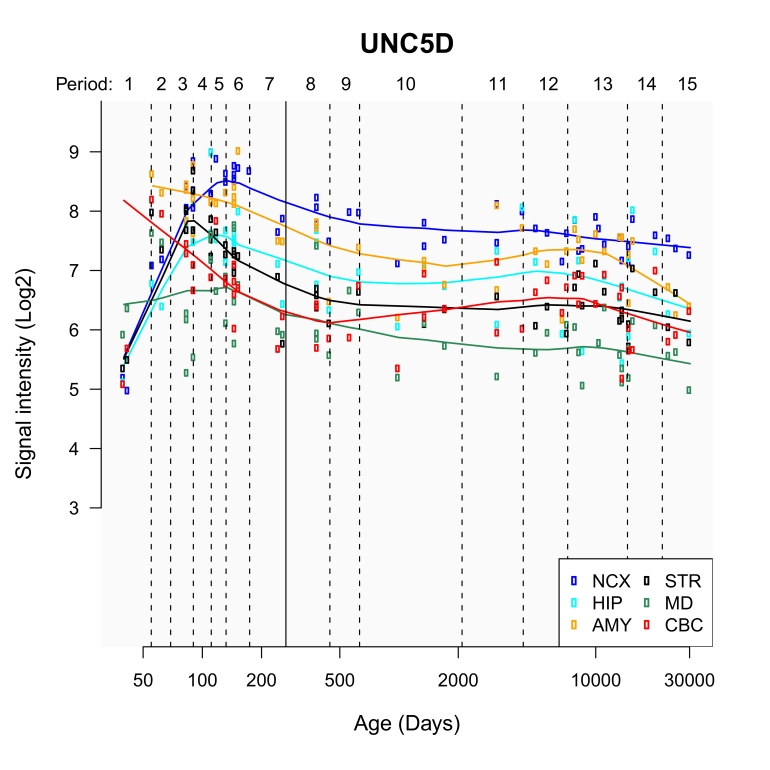

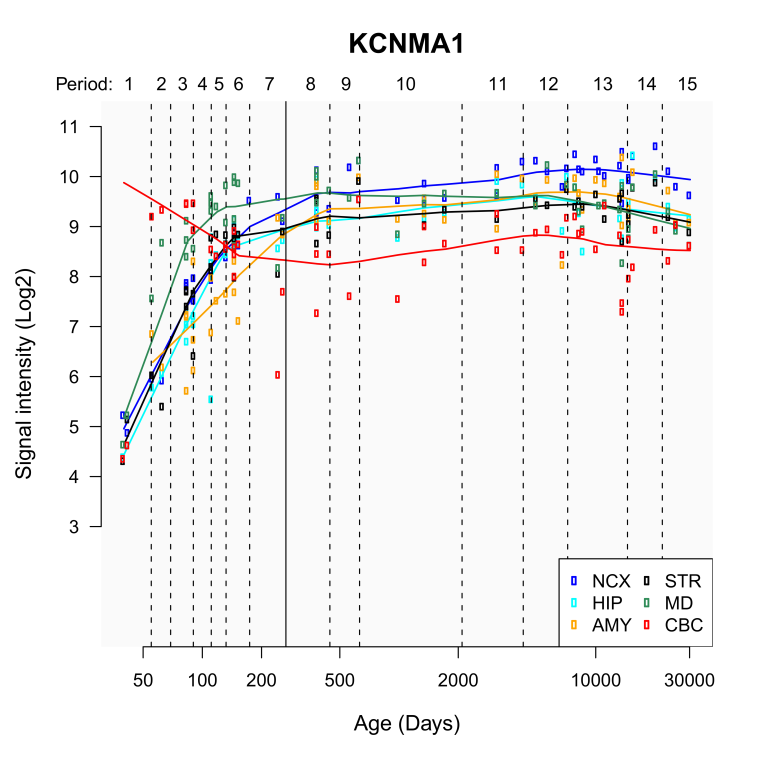

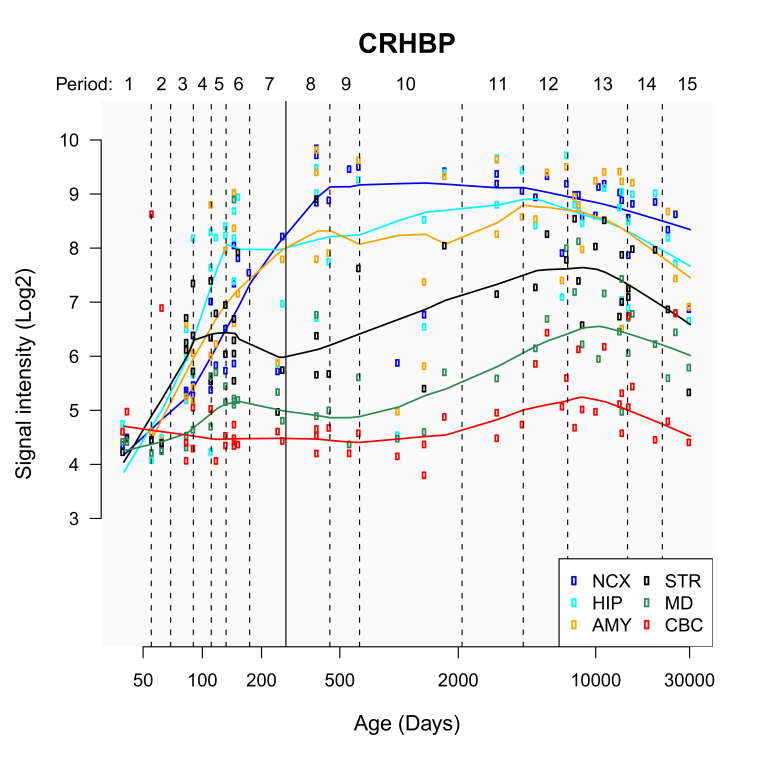


M20

M20

M15

M2
